# 

**Published:** 1888-04

**Authors:** 


MALPOSITIONS OF THE COLON AND CŒCUM. Thomas Walley, M.R.C.V.S. ... 113
NOTE ON TÆNIA ELLIPTICA (CAT). William S. Gottheil, M.D. ... 126
PHYSIOLOGICAL RELATIONS OF GULAR VOCALIZATION IN GROUSE. G. Archie Stockwell, M.D., F.Z.S. ... 127
HOG CHOLERA. Dr. D. E. Salmon ... 136
DESCRIPTION OF SOME SPECIMENS OF PLEURO-PNEUMONIA CONTAGIOSA. A. W. Clement, V.S. ... 151
THE DIAGNOSIS, CLASSIFICATION AND NATURE OF TUMORS. Henry F. Formad, B.M., M.D. 155
THE HARVARD VETERINARY SCHOOL ... 171
EDITORIAL DEPARTMENT ... 177
CASE DEPARTMENT ... 188
CORRESPONDENCE ... 193
OBITUARY ... 196
PROCEEDINGS OF VETERINARY MEDICAL SOCIETIES ... 197
REVIEWS ... 205
BOOKS AND PAMPHLETS RECEIVED ... 207

